# Continuous Program Evaluation to Foster an Environment that Leads to Student Engagement

**DOI:** 10.1093/geroni/igaa057.1758

**Published:** 2020-12-16

**Authors:** Janice Velazquez

**Affiliations:** East Los Angeles College, Los Angeles, California, United States

## Abstract

This presentation explores aspects related to the Gerontology Health Certificate of Achievement program and the progression of their Enrollment Management Plan. It discusses how external forces have influenced changes to the plan during the last five years. Some of the strategies to be addressed are the incorporation of adult education courses to the career path; interdisciplinary collaborations with Health, Theater and Psychology Departments; partnerships with organizations such as American Association Family and Consumer Sciences; Association Gerontology in Higher Education, and private donors. The presenter also covers innovative teaching modalities such as laboratories, research, student club, and online teaching as an attempt to accommodate students’ needs. Lastly, it discusses the incorporation of workforce development data and how it is influenced by national and institutional policies and practices—ultimately creating a healthy environment to promote enrollment growth by strengthening student’s retention and completion. Part of a symposium sponsored by the Community College Interest Group.

